# Postoperative shed autologous blood reinfusion does not decrease the need for allogeneic blood transfusion in unilateral and bilateral total knee arthroplasty

**DOI:** 10.1371/journal.pone.0219406

**Published:** 2019-07-08

**Authors:** YuLiang Miao, WenZhi Guo, LiNa An, WeiWu Fang, Yan Liu, XiaoPing Wang, LiKun An

**Affiliations:** 1 Department of Anesthesiology, Chinese PLA No. 306 Hospital, Beijing, China; 2 Department of Anesthesiology, the seventh center of PLA general hospital, Beijing, China; 3 Department of Anesthesiology, the third center of PLA general hospital, Beijing, China; 4 Department of Orthopaedics, Chinese PLA No. 306 Hospital, Beijing, China; 5 Department of Orthopaedics, Beijing Chao-yang Hospital, Capital Medical University, Beijing, China; Vita Salute University of Milan, ITALY

## Abstract

Postoperative shed autologous blood reinfusion techniques have been used for decades in total knee arthroplasty (TKA), but the effectiveness of this procedure is still a matter of debate. This multicenter retrospective study investigated the medical records of patients who underwent unilateral and bilateral TKA from January 1, 2015 to December 31, 2017 in three hospitals. According to whether postoperative shed autologous blood reinfusion was used, the patients were divided into the control group and the shed autologous blood reinfusion group. The volume of perioperative infusion of red blood cells and plasma, the blood transfusion-related costs, and the postoperative hospital stay were compared between the two groups of patients. A total of 200 unilateral and 74 bilateral TKA were included after successful matching. Among the patients who underwent unilateral TKA, the control group and the shed autologous blood reinfusion group had 95 and 91 patients, respectively, who received allogeneic blood infusion (P = 0.268). There was no significant difference in the number of units of allogeneic red blood cells infused (P = 0.154), while the transfusion-related cost was increased (P<0.001). The same phenomena were observed over the patients underwent bilateral TKA. Shed autologous blood reinfusion does not reduce the need for infusing allogeneic red blood cells. In addition, the procedure increases patient expense and may also lead to an extended postoperative hospital stay.

## Introduction

TKA is currently an important method for the treatment of late and severe knee joint lesions. The surgical operation is complex, involves significant trauma and often leads to large postoperative blood loss. In particular, when both knee joints are replaced simultaneously, the blood loss is substantial. Therefore, the procedure frequently requires a postoperative infusion of allogeneic red blood cells [1, 2]. Although the safety of the transfusion of allogeneic red blood cells has gradually increased, there are still potential risks of allergic reactions, HIV and hepatitis transmission, and bacterial blood contamination[3]. In addition, the current state of clinical blood use in China is dire, as there is a large gap between blood supply and demand. In severe cases, surgery may be suspended due to insufficient blood supply.

At present, the blood-saving measures for TKA include the use of erythropoietin and iron, preoperative collection of autologous blood, intraoperative use of a tourniquet and tranexamic acid and postoperative shed autologous blood reinfusion. The last one has been used for decades, and many studies have been reported, but the conclusions are not consistent. Most of the earlier publications showed that postoperative shed autologous blood reinfusion could save blood use [4, 5], but other recent studies and one meta-analysis suggested the opposite result [6–9]. This difference may be because in the past 30 years, advanced surgical methods and surgical instruments have been developed, and shed autologous blood reinfusion equipment and operational methods have been somewhat improved. As a result, different studies may have adopted different indications for blood transfusion.

Due to the publications of shed autologous blood reinfusion concerning allogeneic blood transfusion after unilateral and bilateral TKA are rare, we designed this study to examine whether shed autologous blood reinfusion can reduce allogeneic blood transfusion and to determine the economic efficiency of this procedure.

## Materials and methods

This multicenter retrospective study was approved by the Ethics Committees of the 306th Hospital of PLA in Beijing, General Hospital of Chinese People’s Armed Police Forces, and People’s Liberation Army General Hospital in Beijing. All three hospitals are large-scale general hospitals. Each hospital has experienced experts at performing TKA. All providers follow the Beijing blood transfusion guidelines and use the same shed autologous blood reinfusion equipment. Postoperative rehabilitation training is also the same.

All patient information was anonymous and only de-identified patient information was used, therefore the need for informed consent was waived by the ethics committee. The inclusion criteria were as follows: age 18–80 years, suffering from osteoarthritis and rheumatoid arthritis, and undergoing the first unilateral or bilateral TKA. The exclusion criteria were as follows: hemorrhage and coagulation disorders, previous history of thrombosis or hemorrhage, liver and kidney function abnormalities, severe lesions of the mental and nervous system, implantation of cardiac pacemaker and in vivo stent.

The time period included in this study was from January 1, 2015, to December 31, 2017. The patients were divided into a control group and a shed autologous blood reinfusion group according to whether the shed autologous blood reinfusion equipment was used. The following data on all patients who underwent unilateral or bilateral TKA were extracted from the electronic medical record databases of the three hospitals: sex; age; American Society of Anesthesiologists (ASA) Physical Status Classification (ASA grade); weight; operation duration; total perioperative blood loss; volume of perioperative allogeneic red blood cells and fresh frozen plasma (FFP) infusion; volume of postoperative shed autologous blood reinfusion; postoperative hospital stay; hemoglobin and hematocrit levels before the surgery, on the day after surgery (Day 0, i.e., 10 h after surgery completion), on the three consecutive days after the surgery (Day 1–3) and prior to discharge.

### Anesthesia

All patients underwent endotracheal intubation, general anesthesia, intravenous and inhalational combined anesthesia or total intravenous anesthesia. Preoperatively, none of the patients had autologous blood collected or was given erythropoietin and iron. Intraoperatively, no controlled hypotension and blood dilution was performed, and no blood coagulant drugs were given.

### Surgical methods and blood transfusion

All patients were placed in a supine position and an anterior midline incision of the knee joint and medial parapatellar approach was used in all cases. All prostheses used were posterior-stabilizing high-flexion rotary platform prostheses (Stryker Inc., Johnson & Johnson) and were fixed with bone cement. A tourniquet was used during all operations, and the tourniquet was used alternately for the operated limb in patients undergoing bilateral knee arthroplasty. The orthopedic surgeon decided whether or not to use postoperative shed autologous blood reinfusion equipment without clear indications and tendencies. A drainage tube was placed on the surface of the prosthesis of each knee joint and was connected to a CBC II ConstaVac type electric negative pressure non-washing postoperative autologous blood collection and transfusion system (Stryker Inc.). In brief, the shed blood was collected into the anticoagulant-free vacuum canister connecting to a reinfusion bag, then the shed blood was transfused through a 40-μm screen filter allowing the elimination of fat particles and other debris. Within 6 h, the autologous blood was directly reinfused as soon as the collection reached 200 ml. After 6 h, the tube was used as an ordinary drainage tube. In the control group, a drainage bag was connected to the drainage tube. The intraoperative blood loss included the blood lost in the aspirator as well as gauze adsorption. The postoperative blood loss included autologous blood reinfusion or the drainage bag blood as well as the exudation absorption from the incision dressing; the sum of both was the total perioperative blood loss.

According to Beijing’s Guidelines for Clinical Blood Transfusion and considering that these patients are elder, and some patients have various comorbidities, both anesthesiologists and orthopedists suggest that more oxygen supply can be provided when the hemoglobin is >10 g/dl, which can reduce or avoid myocardial ischemia and other postoperative complications and may be beneficial for postoperative recovery. Especially in the current medical environment in mainland China ^[^^10^^]^, this approach may be safer for both patients and hospitals. Therefore, the blood transfusion trigger point is set at a hemoglobin of <10 g/dl.

All three hospitals used Beijing’s charging standard for medical treatment. The calculation is based on the RMB exchange rate of 6.28 yuan in January 2018. When ABO and Rh matching, cross-matching, antibody screening, leukocyte filtration and infusion are included, the fee for the infusion of every 1U of allogeneic red blood cells is US$78.79 (from 200 ml whole blood). The fee for the transfusion of every 200 ml of FFP is $14.01. The difference is that every unit of allogeneic red blood cells comes from 500 ml of whole blood in the US, and the average charge is $211, at a cost of $54 for 200 ml. Assuming that the charge for postoperative autologous red blood cell collection and infusion uses Beijing’s fixed price of US$302.55, according to the Beijing standard cost and the US standard cost, we calculated the total perioperative transfusion-related cost for each patient, including shed autologous blood reinfusion, allogeneic red blood cells, and FFP.

### Statistics

This study is retrospective and does not perform randomized grouping; therefore, the key baseline indicators, including sex, age, ASA grade, body weight, operation duration, total perioperative blood loss, preoperative hemoglobin and hematocrit and those at discharge, are not completely consistent. In this study, we used a propensity score matching method to normalize the above indicators [11]. Briefly, the above indicators were substituted into the stepwise logistic regression model, and each patient’s indicator received a propensity score. If the patients with the closest propensity scores in the two groups were matched successfully, they were included in the study. The patients who failed the matching were eventually excluded from the enrolled samples. The specific parameter setting used the nearest neighbor method, 1:1 pairing, and the caliper value was set at 0.2. Once the matching was successful, it was not re-entered.

The primary outcome indicator is the total perioperative infusion volume of allogeneic red blood cells and plasma as well as the transfusion-related costs. Continuous variables are expressed as the mean ± standard error, and an independent sample t test was used. The categorical variables are expressed in frequency and chi-square tests are used. SPSS 19.0 is used for statistical analysis, and the significant difference is set as P<0.05.

## Results

According to the inclusion and exclusion criteria of this study, a total of 448 patients and 182 patients who underwent unilateral and bilateral TKA, respectively, were included. However, some of the medical records were not complete. Moreover, one patient who had a previous gastrointestinal ulcer history and died of massive, uncontrollable gastrointestinal blood loss 10 days after the surgery was excluded. Finally, a total of 357 cases of unilateral TKA were enrolled. Of these, the control group contained 214 patients, and the shed autologous blood reinfusion group contained 143 patients. A total of 158 patients underwent bilateral TKA. Of these, 86 patients were in the control group, and 72 patients were in the shed autologous blood reinfusion group.

After the propensity score matching, there were 100 cases of unilateral TKA and 37 cases of bilateral knee arthroplasty in each group (see Tables 1 and 2). The distribution of preoperative hypertension, diabetes, coronary heart disease and other internal diseases in patients of each group is shown in Table 3. In all cases, no secondary surgery was required due to surgical site infection before discharge. There was no severe deep venous thrombosis or serious anaphylaxis caused by transfusion or other complications such as low-grade DIC.

**Table 1:** Patients’ demographic characteristics before and after propensity score matching, unilateral TKA.

	Overall Cohort			Propensity-Matched Cohort		
	Control	Cell saver		Control	Cell saver	
	(n = 214)	(n = 143)	P	(n = 100)	(n = 100)	P
**Sex (Male/Female)**	42/172	25/118	0.612	18/82	19/81	0.856
**Age (yr)**	63.42±0.68	64.89±0.71	0.147	64.21±1.05	64.72±0.81	0.702
**ASA degree**			0.048			0.943
**I**	7	6		5	4	
**II**	147	113		77	78	
**III**	60	24		18	18	
**Body weight (kg)**	69.69±0.78	67.50±0.85	0.065	69.13±1.05	68.82±1.01	0.832
**Duration of the operation (min)**	136.03±2.17	121.14±2.49	<0.001	125.96±2.99	125.25±3.14	0.870
**Total blood loss (ml)**	864.31±46.11	1159.86±55.71	<0.001	1044.45±47.28	1120.35±71.83	0.378
**Pre-op hemoglobin (g/dl)**	130.38±0.98	126.32±1.04	0.006	130.12±1.41	128.74±1.20	0.457
**Pre-op hematocrit**	39.33±0.27	38.60±0.31	0.080	39.38±0.41	39.02±0.36	0.507
**Discharge hemoglobin (g/dl)**	106.31±0.81	107.15±1.23	0.555	106.68±1.13	107.02±1.61	0.863
**Discharge hematocrit**	32.12±0.24	32.63±0.31	0.198	32.39±0.34	32.50±0.38	0.833

**Table 2:** Patient demographic characteristics before and after propensity score matching, bilateral TKA.

	Overall Cohort			Propensity-Matched Cohort		
	Control	Cell saver		Control	Cell saver	
	(n = 86)	(n = 72)	P	(n = 37)	(n = 37)	P
**Sex (Male/Female)**	14/72	8/64	0.350	5/32	3/34	0.708
**Age (yr)**	63.60±0.83	64.71±0.79	0.342	63.59±1.26	62.43±1.06	0.483
**ASA degree**			0.016			0.239
**I**	0	5		0	2	
**II**	66	49		30	29	
**III**	20	18		7	6	
**Body weight (kg)**	68.78±1.09	70.50±1.30	0.308	70.04±1.87	68.08±1.31	0.394
**Duration of the operation (min)**	239.72±7.04	191.04±4.32	<0.001	212.73±9.31	204.78±5.28	0.461
**Total blood loss (ml)**	1742.71±67.87	2173.26±68.13	<0.001	2087.03±101.37	2109.05±92.81	0.873
**Preoperative hemoglobin (g/dl)**	131.31±1.45	129.03±1.47	0.273	131.59±1.91	129.24±2.14	0.415
**Preoperative hematocrit**	39.64±0.38	39.33±0.48	0.604	39.74±0.54	39.20±0.71	0.547
**Discharge hemoglobin (g/dl)**	99.80±1.36	100.16±1.60	0.865	99.70±1.95	99.60±2.44	0.974
**Discharge hematocrit**	30.30±0.42	30.88±0.56	0.406	30.26±0.58	30.38±0.87	0.908

**Table 3:** Distribution of preoperative internal diseases.

	Unilateral knee joint			Bilateral knee joint		
	Control group n = 100	Autologous blood group n = 100	P value	Control group n = 37	Autologous blood group n = 37	P value
**Hypertension**	33	39	0.3768	14	10	0.3206
**Diabetes**	13	12	0.8307	3	3	1.000
**Bronchitis**	1	2	1.000	2	0	0.473
**Coronary heart disease**	11	17	0.221	4	3	0.700
**Hyperlipidemia**	1	3	0.614	0	2	0.473
**Gastric ulcer**	3	4	1.000	0	1	1.000
**Atrial fibrillation**	1	1	1.000	0	0	/

In patients undergoing unilateral TKA, 95 and 91 patients in the control group and the shed autologous blood reinfusion group, respectively, received an infusion of allogeneic red blood cells, and there was no significant difference between these two groups(P = 0.268). The reinfusion volume in the postoperative shed autologous blood reinfusion group was 536.33±38.54 ml. During the entire perioperative period, there was no significant difference in the infused allogeneic red blood cells between the shed autologous blood reinfusion group and the control group (3.70±0.17 vs 3.29±0.17 units, P = 0.154). Compared to the control group, there was no significant difference in the infused volume of (FFP) by the shed autologous blood reinfusion group (299.50±42.71 vs 338.00±26.47, P = 0.444). On the day of surgery, and on the first day after surgery, the hemoglobin content in the shed autologous blood reinfusion group was increased compared to the control group (P = 0.002 and P = 0.029). There was also no significant difference in the postoperative hospital stay (14.97±0.46 vs 15.58±0.61, P = 0.429), (see Table 4).

**Table 4:** Unilateral TKA transfusion results.

	Control	Cell saver	P
	(n = 100)	(n = 100)	
**Transfused numbers**	95	91	0.268
**Allogeneic RBC transfusion (units)**	3.70±0.17	3.29±0.17	0.154
**FFP transfusion (ml)**	299.50±42.71	338.00±26.47	0.444
**Reinfused RBC volume (ml)**		536.33±38.54	
**Postoperative Day 0 Hb**	108.60±2.30	116.99±1.39	0.002
**Postoperative Day 1 Hb**	103.57±2.29	109.33±1.45	0.029
**Postoperative Day 2 Hb**	104.68±2.59	102.08±2.13	0.442
**Postoperative Day 3 Hb**	100.69±2.30	105.82±2.22	0.126
**Postoperative hospital stay (days)**	14.97±0.46	15.58±0.61	0.429

In patients undergoing bilateral TKA, all 74 patients in both groups received an infusion of allogeneic blood. The volume of postoperative shed autologous blood reinfusion was 1008.38±72.99 ml. During the entire perioperative period, there was no significant difference in the volume of infused allogeneic erythrocytes (7.89±0.44 vs 7.14±0.40, P = 0.207) or (FFP) (556.76±52.64 vs. 683.78±61.21, P = 0.120) between the control group and the shed autologous blood reinfusion group. Compared to the control group, the hemoglobin of the shed autologous blood reinfusion group was higher on the day of surgery (P = 0.023), and the postoperative hospital stay was significantly increased (15.41±0.86 days vs 18.41±0.91 days, P = 0.020); see Table 5.

**Table 5:** Bilateral TKA transfusion results.

	Control	Cell saver	*P*
	(n = 37)	(n = 37)	
**Transfused numbers**	37	37	1.000
**Allogeneic RBC transfusion (units)**	7.89±0.44	7.14±0.40	0.207
**FFP transfusion (ml)**	556.76±52.64	683.78±61.21	0.120
**Reinfused volume of RBC (ml)**		1008.38±72.99	
**Postoperative Day 0 Hb**	105.39±2.63	115.77±3.58	0.023
**Postoperative Day 1 Hb**	105.86±2.59	113.15±3.23	0.113
**Postoperative Day 2 Hb**	103.81±3.58	96.13±3.51	0.143
**Postoperative Day 3 Hb**	98.65±2.36	96.67±3.43	0.657
**Postoperative hospital stay (days)**	15.41±0.86	18.41±0.91	0.020

The cost in the shed autologous blood reinfusion group was significantly higher than the control group, according to both the standard cost in Beijing ($312.50±14.68 vs. $582.03±19.73, P<0.001) and the standard cost in the US ($393.15±20.79 vs $668.04±24.94, P<0.001). In patients undergoing bilateral TKA, the cost of the shed autologous blood reinfusion group was also increased significantly compared to the control group ($660.80±37.30 vs $1215.18±34.59 based on the standard cost in Beijing, P<0.001, and $816.40±47.77 vs $1391.93±47.06 based on the standard cost in the US, P<0.001).

## Discussion

In this study, we investigated whether the use of shed autologous blood reinfusion equipment after unilateral or bilateral TKA can reduce the need for allogeneic red blood cell infusion and improve economic efficiency. The results suggest that the procedure does not reduce the demand for allogeneic red blood cells and plasma. In fact, it increases the economic burden on patients.

At present, approximately 300,000 TKA operations are performed each year in China. As one of the important methods of blood management, shed autologous blood reinfusion technology has been applied to TKA for nearly 30 years, and its materials and application methods have been continuously improved. There are many factors influencing blood loss in TKA, which mainly include surgical approaches, usage of tourniquets , placement of drainage tubes, the number and location of the drainage tubes, the type of equipment for shed autologous blood reinfusion, usage of postoperative negative pressure drainage and blood transfusion indications [9, 12].

Some reports suggested that shed autologous blood reinfusion could reduce the demand for allogeneic blood [4, 5]. Similar to this conclusion is the meta-analyses by Carless et al [13] and Hong et al [14], which included 18 studies that covered from 1991 to 2008 and 15 papers that spanned from 1991 to 2014, respectively. Both meta-analyses concluded that shed autologous blood reinfusion could significantly reduce the infusion rate and volume of allogeneic red blood cells. However, other studies indicated that shed autologous blood reinfusion was ineffective [6–8, 15, 16]. This view was discussed in the meta-analysis published in 2015 by Bodegom-Vos et al [9]. Their study included 25 papers spanning from 1991 to 2014. The combined analysis of the 25 papers revealed that autologous blood reinfusion had a blood-saving effect. However, after the 25 papers were stratified, the conclusion of the subgroups of 1991–1999 and 2000–2010 was that it could save blood use, whereas the higher quality papers published in the subgroup of 2010–2014 indicated that shed autologous blood reinfusion did not have a blood-saving effect.

Studies have shown that the average total blood loss in unilateral knee arthroplasty can be as much as 750–1500 ml [1, 2, 17, 18]. Earlier literature suggested that the overall blood loss was still as high as 1518 ml on average even if a tourniquet was used during the entire procedure [19]. In addition to the visible blood loss, hidden blood loss may be even more severe [18]. A study by Rosencher et al [17] showed that the visible blood loss in unilateral TKA was 800 ml, while the hidden blood loss was as high as 1934 ml. Correspondingly, the allogeneic blood transfusion rate reached 22–87% [2, 20–22], and the average blood transfusion was 2 units [20]. Some studies [16] suggested that the blood loss in unilateral or bilateral knee arthroplasty can be as high as 1292 and 2043 ml. Similar to this result, the total blood loss caused by unilateral and bilateral knee arthroplasty in this study exceeded 1000 and 2000 ml, respectively, and the transfusion rates were as high as 91–95% and 100%, respectively. In the present study, the total volumes of reinfusion in the unilateral and bilateral TKA were 536.33±38.54 ml and 1008.38±72.99 ml, respectively. There were no severe adverse reactions.

In this study, we included 200 cases of unilateral and 74 cases of bilateral TKA from 3 large hospitals in Beijing. The results suggested that shed autologous blood reinfusion could not reduce the demand for allogeneic red blood cells (P = 0.154 and P = 0.207); instead, it significantly increased economic costs (P<0.001 and P<0.001). We noted that there was no significant difference in the total volume of infused allogeneic red blood cell after unilateral and bilateral knee arthroplasty. The shed autologous blood reinfusion groups were transfused with 536.33 ml and 1008.38 ml autologous blood within 6 h after the surgery, respectively. Interestingly, in unilateral TKA, the hemoglobin in the shed autologous blood reinfusion group was significantly increased compared to the control group on the day of surgery (108.60±2.30 vs 116.99±1.39, P = 0.002) and the following day (103.57±2.29 vs 109.33±1.45, P = 0.029). Starting from postoperative day 3, there was no significant difference in Hb level between the two groups. There was a similar trend in bilateral TKA. On the day of surgery, the difference in the level of hemoglobin between the two groups was significant (105.39±2.63 vs. 115.77±3.58, P = 0.023). Starting from the second day after the surgery, the level of hemoglobin became consistent (P = 0.113). We speculate that this may be because at the time of allogeneic red blood cells infusion, the shed autologous blood reinfusion groups were infused with a large volume of autologous blood; therefore, the hemoglobin level was higher for a short period of time after the surgery[15].

Few studies have evaluated the economic efficiency of shed autologous blood reinfusion equipment, and the conclusions are conflicting. A study by Thomas [23] indicated that shed autologous blood reinfusion could significantly reduce the demand for allogeneic blood, but on average, the medical cost for each patient was increased by 113 pounds. On the contrary, a meta analysis by Han et al[24] demonstrated that the application of tranexamic acid has blood-sparing and cost-benefit properties in theses patients. Unfortunately, tranexamic acid was not used in the three hospitals above because of clinical habit which was verified the efficacy of blood sparing. Meanwhile, some negative aspects exists such as the potential risk of clotting since the blood goes through artificial surface including ConstaVac.

Interestingly, the length of postoperative hospitalization among patients undergoing bilateral TKA was significantly increased (15.41±0.86 vs 18.41±0.91, P = 0.020) in this study. This may be because the main result of this study was that shed autologous blood reinfusion could not reduce the need for allogeneic blood in TKA [25]. Moreover, if shed autologous blood reinfusion indirectly leads to an extended postoperative hospitalization, it may bring a hidden increase in patient costs, and the specific values such as caregiving costs are difficult to estimate.

The strengths of this study are as follows: 1. The study included three large comprehensive hospitals in Beijing, and each of them had over 1000 beds. Moreover, there was consistency in the anesthesia methods, application of tourniquets, etc, and the clinical bias was small. 2. Since 2000, the number of published RCT studies on shed autologous blood reinfusion after TKA has been limited. Only two papers have over 100 cases in each group [23, 25], and both of these were unilateral TKA. In this study, 200 and 74 cases of unilateral and bilateral TKA, respectively, were investigated; no similar studies have been reported so far. 3. In this study, we investigated the volume and the cost of the infusion of FFP. No such investigation has been reported in similar studies.

The limitations of this study are as follows: 1. Considering that the patients in this study were elder, regardless of whether the patient had symptoms such as hypotension and circulatory instability, in practice, Hb <10 g/dl was always used as a transfusion indication. In other studies published earlier, the indications for blood transfusion were mostly Hb <9 g/dl and clinical symptoms [4–6, 15, 26]. If the blood transfusion indication in this study was more rigorous, it may have led to different outcomes. 2. Since this is a multicenter retrospective study, it is still not possible to completely exclude other potential clinical biases that may affect the overall outcome.

## Conclusion

In conclusion, the results of this study suggest that postoperative shed autologous blood reinfusion cannot reduce the need for allogeneic blood in unilateral and bilateral TKA. Instead, shed autologous blood reinfusion can significantly increase transfusion-related costs. In addition, autologous transfusion may lead to an extended postoperative hospital stay. However, since this study is retrospective in nature, it is necessary to perform a well-designed large-scale multicenter randomized controlled trial in the future for further investigation.

## References

[1] Mirzatolooei F, Tabrizi A, Gargari MM. A Comparison of the Postoperative Complications between Two Drainage Methods after Total Knee Arthroplasty. Arch Bone Jt Surg. 2018; 6(1): 47–51. 29430495 PMC5799600

[2] Oberhofer D, Sakic K, Jankovic S, Tonkovic D, Vrgoc G. [How to improve perioperative blood management in patients undergoing total hip or knee replacement surgery?]. Lijec Vjesn. 2012; 134(11–12): 322–327. 23401978

[3] Carson JL, Guyatt G, Heddle NM, Grossman BJ, Cohn CS, Fung MK, et al Clinical Practice Guidelines From the AABB: Red Blood Cell Transfusion Thresholds and Storage. Jama. 2016; 316(19): 2025–2035. Epub 2016/10/13. 10.1001/jama.2016.9185 . 27732721

[4] Atay EF, Guven M, Altintas F, Kadioglu B, Ceviz E, Ipek S. Allogeneic blood transfusion decreases with postoperative autotransfusion in hip and knee arthroplasty. Acta orthopaedica et traumatologica turcica. 2010; 44(4): 306–312. Epub 2011/01/22. 10.3944/AOTT.2010.2417 21252608

[5] Blatsoukas KS, Drosos GI, Kazakos K, Papaioakim M, Gioka T, Chloropoulou P, et al Prospective comparative study of two different autotransfusion methods versus control group in total knee replacement. Archives of orthopaedic and trauma surgery. 2010; 130(6): 733–737. Epub 2010/02/19. 10.1007/s00402-010-1062-y . 20165861

[6] Cip J, Widemschek M, Benesch T, Waibel R, Martin A. Does single use of an autologous transfusion system in TKA reduce the need for allogenic blood?: a prospective randomized trial. Clinical orthopaedics and related research. 2013; 471(4): 1319–1325. Epub 2012/12/12. 10.1007/s11999-012-2729-1 . 23229426 PMC3586038

[7] Dutton T, De-Souza R, Parsons N, Costa ML. The timing of tourniquet release and 'retransfusion' drains in total knee arthroplasty: A stratified randomised pilot investigation. The Knee. 2012; 19(3): 190–192. Epub 2011/03/29. 10.1016/j.knee.2011.02.013 . 21440444

[8] So-Osman C, Nelissen RG, Koopman-van Gemert AW, Kluyver E, Poll RG, Onstenk R, et al Patient blood management in elective total hip- and knee-replacement surgery (Part 1): a randomized controlled trial on erythropoietin and blood salvage as transfusion alternatives using a restrictive transfusion policy in erythropoietin-eligible patients. Anesthesiology. 2014; 120(4): 839–851. Epub 2014/01/16. 10.1097/ALN.0000000000000134 . 24424070

[9] van Bodegom-Vos L, Voorn VM, So-Osman C, Vliet Vlieland TP, Dahan A, Koopman-van Gemert AW, et al Cell Salvage in Hip and Knee Arthroplasty: A Meta-Analysis of Randomized Controlled Trials. The Journal of bone and joint surgery American volume. 2015; 97(12): 1012–1021. Epub 2015/06/19. 10.2106/JBJS.N.00315 . 26085536

[10] Chinese doctors are under threat. Lancet. 2010; 376(9742): 657 Epub 2010/08/31. 10.1016/S0140-6736(10)61315-3 . 20801385

[11] Guo S, MW F. Propensity score analysis: Statistical methods and applications. CA, Sage: Thousand Oaks 2009.

[12] Martin A, von Strempel A. Transfusion of autologous blood from reinfusion systems in total knee arthroplasty. International orthopaedics. 2006; 30(6): 541–544. Epub 2006/08/10. 10.1007/s00264-006-0127-6 . 16896876 PMC3172733

[13] Carless PA, Henry DA, Moxey AJ, O'Connell D, Brown T, Fergusson DA. Cell salvage for minimising perioperative allogeneic blood transfusion. Cochrane Database Syst Rev. 2010; 14(4). 10.1002/14651858.CD001888.pub4 PMC4163967 20393932

[14] Hong KH, Pan JK, Yang WY, Luo MH, Xu SC, Liu J. Comparison between autologous blood transfusion drainage and closed-suction drainage/no drainage in total knee arthroplasty: a meta-analysis. BMC musculoskeletal disorders. 2016; 17: 142 Epub 2016/08/02. 10.1186/s12891-016-0993-z . 27476506 PMC4968028

[15] Abuzakuk T, Senthil Kumar V, Shenava Y, Bulstrode C, Skinner JA, Cannon SR, et al Autotransfusion drains in total knee replacement. Are they alternatives to homologous transfusion? International orthopaedics. 2007; 31(2): 235–239. Epub 2006/06/09. 10.1007/s00264-006-0159-y . 16761149 PMC2267563

[16] Kim DH, Lee GC, Lee SH, Pak CH, Park SH, Jung S. Comparison of Blood Loss between Neutral Drainage with Tranexamic Acid and Negative Pressure Drainage without Tranexamic Acid Following Primary Total Knee Arthroplasty. Knee surgery & related research. 2016; 28(3): 194–200. Epub 2016/09/07. 10.5792/ksrr.2016.28.3.194 . 27595072 PMC5009043

[17] Rosencher N, Kerkkamp HE, Macheras G, Munuera LM, Menichella G, Barton DM, et al Orthopedic Surgery Transfusion Hemoglobin European Overview (OSTHEO) study: blood management in elective knee and hip arthroplasty in Europe. Transfusion. 2003; 43(4): 459–469. 12662278 10.1046/j.1537-2995.2003.00348.x

[18] Sehat KR, Evans RL, Newman JH. Hidden blood loss following hip and knee arthroplasty. Correct management of blood loss should take hidden loss into account. J Bone Joint Surg Br. 2004; 86(4): 561–565. 15174554

[19] Lotke PA, Faralli VJ, Orenstein EM, Ecker ML. Blood loss after total knee replacement. Effects of tourniquet release and continuous passive motion. The Journal of bone and joint surgery American volume. 1991; 73(7): 1037–1040. 1874765

[20] Gombotz H, Rehak PH, Shander A, Hofmann A. Blood use in elective surgery: the Austrian benchmark study. Transfusion. 2007; 47(8): 1468–1480. 10.1111/j.1537-2995.2007.01286.x 17655591

[21] Rao VK, Dyga R, Bartels C, Waters JH. A cost study of postoperative cell salvage in the setting of elective primary hip and knee arthroplasty. Transfusion. 2012; 52(8): 1750–1760. Epub 2012/02/22. 10.1111/j.1537-2995.2011.03531.x . 22339139 PMC3360121

[22] Sinclair KC, Clarke HD, Noble BN. Blood management in total knee arthroplasty: a comparison of techniques. Orthopedics. 2009; 32(1): 19 19226044 10.3928/01477447-20090101-21

[23] Thomas D, Wareham K, Cohen D, Hutchings H. Autologous blood transfusion in total knee replacement surgery. Br J Anaesth. 2001; 86(5): 669–673. 10.1093/bja/86.5.669 11575343

[24] Han X, Gong G, Han N, Liu M. Efficacy and safety of oral compared with intravenous tranexamic acid in reducing blood loss after primary total knee and hip arthroplasty: a meta-analysis. BMC musculoskeletal disorders. 2018; 19(1): 018–2358. 10.1186/s12891-018-2358-2 PMC6278110 30509227

[25] So-Osman C, Nelissen RG, Eikenboom HC, Brand A. Efficacy, safety and user-friendliness of two devices for postoperative autologous shed red blood cell re-infusion in elective orthopaedic surgery patients: A randomized pilot study. Transfus Med. 2006; 16(5): 321–328. Epub 2006/09/27. 10.1111/j.1365-3148.2006.00705.x . 16999754

[26] Cheng SC, Hung TS, Tse PY. Investigation of the use of drained blood reinfusion after total knee arthroplasty: a prospective randomised controlled study. J Orthop Surg. 2005; 13(2): 120–124. 10.1177/230949900501300203 16131672

